# Don’t Shut up the Windows

**Published:** 1891-07

**Authors:** 


### DON'T SHUT UP THE WINDOWS.

Where the body is not overheated the draught caused by the ordinary incoming of air through an open window will do infinitely less harm than the impure air caused by closed windows. The way to enjoy pure air in cold weather is to turn on the heat when the room gets cold, not to shut up the windows. If the room becomes too warm, don't turn off the heat, but open the windows. By this means a person who knows any thing about ventilation can have an equable, summer-like atmosphere about him all winter long. The necessity of open windows is doubly apparent where tobacco smoke is indulged in, as the smoke is dangerous to the breathing apparatus and makes it liable to lung troubles.

